# Sick but satisfied: The impact of life and health satisfaction on choice between health scenarios^☆^

**DOI:** 10.1016/j.jhealeco.2013.04.002

**Published:** 2013-07

**Authors:** Paul Dolan, Georgios Kavetsos, Aki Tsuchiya

**Affiliations:** a Department of Social Policy, London School of Economics, Houghton Street, London WC2A 2AE, UK; b Department of Economics, University of Sheffield, Sheffield S1 4DT, UK; c School of Health and Related Research, University of Sheffield, S1 4DA, UK

**Keywords:** EQ-5D, Life satisfaction, Health valuation

## Abstract

Preference elicitation methods require respondents to predict the impact a change in health might have on their future selves. The focus on the change in health is at the possible expense of other experiences of life once in that health state. We analyse personal preferences to a pairwise choice task involving trade-offs between quality and length of life, where satisfaction levels with life or health are introduced in the description of the health states. We find that a health scenario including low levels of satisfaction increases the likelihood of preferring to die sooner in full health, whereas scenarios including high levels of satisfaction increase the likelihood of preferring to live for longer in poor health. The differences highlight the sensitivity of preferences to what is described in health states and therefore show the importance of on-going discussions about precisely what respondents should be asked to consider in preference elicitation studies.

## 1 Introduction

Gains in quality-adjusted life years (QALYs) are a well-established way of capturing the benefits of health care in a single metric that combines quality of life with length of life. They are currently used by many health technology assessment (HTA) agencies around the world – most notably the National Institute for Health and Clinical Excellence (NICE) in the UK – to assist in the allocation of scarce health care resources.

A critical question in the valuation of QALYs is how best to describe health in such a way that allows comparisons across a broad range of health conditions. One of the most widely used health state descriptive systems (and the one recommended by NICE) is the EQ-5D, which describes health in terms of three levels (essentially no, moderate, and severe problems) of five dimensions (mobility, self-care, usual activities, pain/discomfort, and anxiety/depression) (Brooks, 1996).

Preferences over composite EQ-5D health states are often then elicited from the general public, and sometimes from patients, using the standard gamble (SG) and/or time trade-off (TTO) method (Torrance, 1986). Many HTA agencies recommend the use of public values; that is, preferences over hypothetical health states. In principle, these methods place all health states on an interval scale between 0 (for dead) and 1 (for full health), thus allowing for the calculation of QALYs – see Dolan (1997) for an EQ-5D ‘tariff’ based on TTO valuations from the UK general public and Tsuchiya et al. (2006) for a comparison of methods used to elicit preferences.

Typical preference elicitation studies present the respondents with hypothetical health states described, for instance by EQ-5D, with no further information. In particular, they give no indication of how the experience of living in the health state is to be perceived (Dolan and Kahneman, 2008). This raises the question of whether preference-based valuation studies could more fully communicate the longer-term impacts. So this study seeks to elicit ‘more informed’ preferences obtained via a TTO that incorporates various levels of satisfaction with life or health alongside the standard health state descriptors.

Measures of satisfaction with life or health have been widely used to assess subjective well-being (Sumner, 1996; Diener et al., 1999). The measures have been shown to be associated with many life events and circumstances in ways that would be predicted (Dolan et al., 2008) and the responses correlate well with other measures, such as the reports of other people (Sandvik et al., 1993) and brain activity (Sutton and Davidson, 1997; Urry et al., 2004). Incorporating this information in a health state description offers a concise and informative way of communicating the experience of living in a hypothetical state of health. It is important then to see what effect including satisfaction with life or health in the description of a health state scenario – alongside its EQ-5D health state and duration – has on the preferences people express over it.

Our approach differs from studies focusing specifically on disparities attributed to framing effects (Tversky and Kahneman, 1981) due, for example, to labelling condition-specific preference-based measures (Rowen et al., 2012) and framing of SG and TTO scenarios in terms of losses or gains (Blumenschein and Johannesson, 1998; Stalmeier and Bezembinder, 1999). Our paper may be interpreted as exploring the effect of adding a “bolt-on” item on to the EQ-5D that provides additional information on the hypothetical scenario being valued – for example, Krabbe et al. (1999), Wolfs et al. (2007) and Yang et al. (forthcoming) include additional dimensions in the EQ-5D description system, such as a cognitive or ‘sleep’ dimension. In contrast to these papers, our study “bolts-on” important overall experiences of a person's life.

Against this background, the primary question in this paper is: are health state preferences influenced by satisfaction levels in those states? In addition, an individual's current health satisfaction and life satisfaction might also be important explanatory variables in their preferences. So we additionally ask: are health state values, which contain satisfaction levels, influenced by the respondent's own satisfaction level?

The next section describes the data and methods used. Section 3 presents the results of the analysis. We find that a health scenario that includes low levels of satisfaction increases the likelihood of preferring to die sooner in full health. Similarly, scenarios that include high levels of satisfaction significantly increase the likelihood of preferring to live for a longer time despite being in poor health. These preferences are not significantly affected by the respondent's own health or life satisfaction. Section 4 puts these findings in context and offers directions for future research.

## 2 Data and methods

The data used in this study are part of a larger UK methodological project in preparation for the re-valuation of the set of values for the EQ-5D (for further details of the wider study, including the recruitment of the sample, see Tsuchiya and Mulhern, 2011). Our data consist of respondents’ own, personal, preferences to a series of pairwise choices asking the respondent to imagine the possibility of not being in a perfect state of health – see Dolan et al. (2003) for a framework of different perspectives when eliciting preferences. These are of the following kind:

*Scenario A*: You live in health state *H* with satisfaction *S* for 5 years, and then die.
*Scenario B*: You live in full health for 3 years, and then die.

The durations are fixed at 5 years for scenario A and 3 years for scenario B. These durations are arbitrarily selected and do not necessarily satisfy the constant proportional time trade-off assumption – postulating that respondents trade-off the same fraction of life years irrespective of the duration of the health state – for which the empirical evidence is somewhat mixed (Dolan and Stalmeier, 2003; Craig, 2009).

Three hypothetical health states are used from the EQ-5D-5L, where each consists of just one problem: ‘unable to walk’, ‘extreme pain’, and ‘extreme depression’ (Table A1 lists all five EQ dimensions and levels of severity). These are combined with one of the four satisfaction states – ‘high life satisfaction’ (High LS), ‘high health satisfaction’ (High HS), ‘low life satisfaction’ (Low LS), ‘low health satisfaction’ (Low HS) – and a situation described as ‘learnt to live’ with the health condition. ‘High/low’ were chosen as there is no ambiguity in which of the two is better. Table 1 summarises the overall design.

Furthermore, all respondents of the wider project also answered pairwise choice questions based on a typical TTO scenario, where scenario A was of the form: “You live in health state *H* for *T* years, then die”. We incorporate these data and estimate the probability of respondent *i* choosing to live for 3 years in full health (scenario B). Trivially, in order to attribute any differences in preferences to the introduction of satisfaction levels, all other determinants influencing preferences must be held fixed. For the ‘typical’ scenarios we thus only consider those respondents with the same time trade-offs to be incorporated to our primary dataset – i.e. poor health for *T* = 5 years and full health for 3 years.

For each of the three EQ-5D health dimensions included in this study – i.e. ‘unable to walk’, ‘extreme pain’, and ‘extreme depression’ – we separately estimate the following probit model:

(1) $$P{\left(B\right)}_{i}=\beta_{0}+\sum_{j}[\beta_{1j}{\left(H\times S\right)}_{j}]+\beta_{2}DEMO_{i}+\beta_{3}HEALTH_{i}+\beta_{4}SWB_{i}+\epsilon_{i}$$

where (*H* × *S*) denotes the combination of a health state (*H*) with a level of satisfaction (*S*) under scenario A. In this specification, the ‘typical’ scenario serves as the ‘neutral’ reference category within each health dimension. Regression estimates resulting from this estimation will hence capture any changes in preferences attributed to the introduction of satisfaction levels in the scenario for a given health dimension. The subscript *j* represents the number of multi-attribute health states within a grouped EuroQol dimension. *DEMO* represents a set of demographic variables available for the respondent – these are gender, age, age squared, marital status, employment status, and education level. *HEALTH* is a set of 15 dummy variables capturing respondents’ own state of health obtained from their completion of the EQ-5D-5L (Herdman et al., 2011). *SWB* represents subjective well-being, measured by a set of self-reported satisfaction with life or health variables, each on a 0–10 scale, with 0 denoting ‘not at all satisfied’ and 10 denoting ‘completely satisfied’. As respondents face multiple scenarios of health state–satisfaction combinations, we allow for the correlation of the error term within, but not between, individuals by estimating robust standard errors clustered at the individual level.

## 3 Results

Table 2 describes the data. Of the 645 respondents who answered the relevant questions to this paper, just under half the sample are male, just over half are married and employed, just over a third have a degree. The average age is 42. About 73%, 87%, and 71% of the respondents do not have any problems with mobility, self-care, and performing usual activities, respectively. The corresponding proportions for pain/discomfort and anxiety/depression are about 45% and 56%, respectively. The proportion of those reporting the best state in EQ-5D-5L (i.e. 11111) is 31.6%.

Average own health satisfaction (*SWB*_H_) and life satisfaction (*SWB*_L_) are 6.4 and 6.3, with standard deviations of 2.6 and 2.5, respectively. The distribution of these two measures is quite similar too: their correlation coefficient is 0.67. Given this high correlation, inclusion of both *SWB*_H_ and *SWB*_L_ in Eq. (1) will lead to problems of multi-collinearity. Results presented here control only for *SWB*_L_, although the implications of the results do not differ substantially if controlling for *SWB*_H_ instead.

In addition, following Dolan and Metcalfe (2011), we merge levels of *SWB*_H_ and *SWB*_L_ into the following categories: ‘low’ if 0–5; ‘medium’ if 6–7; ‘high’ if 8–9; and ‘very high’ if 10. As noted in their study, the justification for separating those scoring 10 (the ‘tens’) is because they are a little different from other respondents in ways that might not be expected – they tend to be older and less healthy, for example.

Pooling all observations from Table 1 (*N* = 2128), the proportion of respondents facing these scenarios choosing to live for 3 years in full health (scenario B) is 70.1%, ranging from 53.1% (for ‘unable to walk and high LS’) to 90.1% (for ‘extreme pain and low HS’). Preferences towards the full health scenario for each combination of health-satisfaction states are presented in Table 3.

We now turn to the results of the regression analysis, comparing health state scenarios without any information on satisfaction with life or health (i.e. a ‘typical’ TTO scenario) to comparable health state scenarios but which contain information on satisfaction. Table 4 reports marginal effects coefficients resulting from the probit estimation of Eq. (1). We only report statistically significant estimates at the 5% level or higher for the remaining controls.

For ‘unable to walk’ (column 1), the probability of choosing the full health scenario significantly increases by 23% when respondents face a low HS. In contrast, when this level of severity is combined with either high HS or LS, the probability of choosing the full health scenario decreases by about 12 and 14%, respectively. This implies that respondents prefer to cope with the extreme level of severity of this health dimension for a longer time period when they are more satisfied with their health/life. Learning to live with the condition has no statistically significant effect on preferences. This seems to suggest that individuals anticipate coming to terms with the mobility problem even when this additional information is not given.

For ‘extreme pain’ (column 2), the addition of low HS does not have a significant effect on preferences, suggesting that low HS is in line with what people associate with extreme pain. In accordance with the results for the mobility dimension, the presence of high HS or LS reduces the probability of choosing the full health scenario by 29 and 32%, respectively. The presence of ‘learnt to live’ also reduces the probability of choosing the full health scenario by about 32%. In other words, individuals are not anticipating learning to live with pain when it is not indicated.

For ‘extreme depression’ (column 3), the effect of ‘high HS’ and ‘learnt to live’ is similar to the corresponding effects of the other two health dimensions, reducing the probability of choosing full health by about 30%.

Looking across the columns, the relative ordering between high LS and high HS is consistent, but the relative ordering of learnt to live is not. This may be interpreted to suggest that the meaning and the value of learning to live with a health condition depend heavily on the state. Own satisfaction levels with life (*SWB*_L_) do not appear to have a statistically significant effect on respondents’ preferences.

Turning to demographic variables for ‘extreme pain’ (column 2) preference towards the full-health scenario significantly increases with age, a below-degree education, the retired, and among those out of employment due to long-term sickness. A below-degree education has a similar effect towards the preference for full health for ‘extreme depression’ (column 3). Here, in addition, those taking care of the home are less likely to choose full health (i.e. prefer on average to live longer being depressed rather than a shorter time in full health).

The effect of respondents’ own health in EQ-5D on health state preferences is interesting to note here. On average, individuals valuing ‘extreme pain’ and ‘extreme depression’ tend to opt for the full health scenario if they already have problems of extreme mobility (level 5) and slight problems of performing usual activities (level 2), respectively. Notably, respondents with existing problems in pain and depression prefer living longer when faced with states of ‘extreme pain’ and ‘extreme depression’, controlling for satisfaction in these states.

## 4 Discussion

This study seeks to answer whether preferences over health scenarios of differing quality of life and duration are influenced by satisfaction levels introduced in those states. Satisfaction with life or health matters to individuals’ well-being, and incorporating this information in a health state offers a concise and informative communication of the experience of living in a hypothetical poor state of health. In addition, we examine whether health state values which contain satisfaction levels are influenced by the respondent's own satisfaction level. The answer to the first hypothesis is ‘very much so’, and to the second ‘not very likely to be’.

A scenario that contains low health satisfaction leads to a significant increase in the likelihood of preferring to die sooner in full health. A notable exception to this finding is the health state associated with ‘extreme pain’, where the addition of low HS does not have a statistically significant effect on preferences. This could be explained by respondents assuming that extreme pain is associated with low HS in the first place. Similarly, a scenario that contains a high satisfaction with either health or life leads to a significant increase in the likelihood of preferring to live for a longer time in poor health rather than a shorter time in full health.

In contrast, it seems that own life satisfaction of the respondent is generally not influencing health state preferences. Repeating the analyses with own health satisfaction instead does not alter this conclusion. Generally speaking, demographic variables are seldom statistically significant.

On average, respondents facing states of ‘extreme pain’ and ‘extreme depression’ prefer living for longer in those states when they themselves have existing problems on the pain and depression dimensions. A plausible explanation for this tendency might be the belief that extreme pain or depression which they are asked to value cannot be much worse than the existing level of pain or depression they are experiencing – or the fact that having had some problems with those health conditions already, they will be able to cope with even more severe cases of these (i.e. a “how much worse can it be?” effect).

The results found in this study could potentially be attributed to adaptation. Typically, though not always, our levels of *SWB*, such as life satisfaction, will adapt to changes in health (Riis et al., 2005; Dolan and Kahneman, 2008). It seems that there is adaptation to some forms of disability (Oswald and Powdthavee, 2008; Pagán-Rodríguez, 2010) and heart problems (Wu, 2001), but not to mental health problems (Dolan et al., 2011). Bradford and Dolan (2010) use satisfaction data to explain adaptation in terms of the weights attached to different domains of life (e.g. health, work, leisure), which are adjusted following changed circumstances in order to maintain overall life satisfaction. Thus, a scenario containing either high health or life satisfaction could indirectly reflect a degree of adaptation.

This study does not come without limitations. First, it focuses on just three of the five dimensions of the EQ-5D – mobility, pain/discomfort and anxiety/depression. Second, it only assesses these states at their corresponding extreme levels of severity. Third, the duration of the scenarios was held constant. Furthermore, the effect of providing these additional information in health state valuation exercises is unlikely to be additive. This is in line with what was observed in a bolt-on study that conducted a valuation of EQ-5D with an additional dimension on sleep problems; Yang et al. (forthcoming) find interactions between the main dimensions of EQ-5D and the additional dimension. In our study, we do not have enough comparisons across enough scenarios to make any firm claims about the robustness of the findings offered here to other combinations. Without question there is need for more research in incorporating the experience of living in poor health for valuation of health states. Future research could, for example, consider expanding the dimensions, levels of severity, and duration of scenarios studied here, and accounting for possible interactions amongst these.

We also need more attempts directly incorporating information on adaptation in the valuation of health states. Use of ‘adaptation exercises’ has been suggested (Damschroder et al., 2005, 2008) and these could potentially be developed even further. Using this method, McTaggart-Cowan et al. (2011) have recently attempted to refine TTO preferences, where people valuing certain health states are presented with audio-recordings of patients explaining how they got used to live in those states. The information on adaptation is found to positively influence the valuation of these health states.

Arguably though, adaptation exercises do not come without limitations of their own. The immediate question that arises is whose information should be included, as surely not all patients adapt to the same degree to a certain health condition. Moreover, including information on specific patient experience would compromise the generic nature of instruments such as the EQ-5D. Furthermore, compared to a ‘simple’ TTO or pairwise choice, the complexity of adaptation exercises means that individuals must devote a substantial amount of time and attention in order to digest the full information provided (e.g. by listening to audio-recordings).

Preferences could also potentially be elicited from people who have experienced the health state in question, or know someone who has experienced or is experiencing the state. The obvious challenge with this approach then is identifying and sampling those people with the most appropriate levels of experience of a given health state, and it is not clear where ‘inexperienced’ stops and ‘experienced’ starts (Dolan, 1999). In addition, there is the concern that those who have experienced the state for long enough may then treat the full health scenario of the TTO as ‘becoming’ fully healthy instead of ‘being’ fully healthy.

A further alternative is to consider using measures of subjective well-being (such as life satisfaction) directly to make health-related valuations and inform policy decisions (Dolan et al., 2009; Dolan, 2011). For example, Dolan et al. (2012) estimate the impact of dimensions of the SF-6D on life satisfaction and show the biggest decrements are associated with mental health. Consistent with this, Graham et al. (2011) show that anxiety/depression in the EQ-5D has the biggest effect on life satisfaction in a Latin American sample. Similar conclusions on dimensions of both the EQ-5D and the SF-6D are reached using patient data (Mukuria and Brazier, 2013). Crucially, these studies are at odds with the relative impact of different dimensions of health on people's preferences, where problems with mobility feature much more prominently (Brazier et al., 2002; Dolan, 1997), but are not free of limitations of their own – for example, valuations differ depending on the measure of subjective well-being used (Powdthavee and van den Berg, 2011).

Adaptation processes naturally raise normative issues about whether, when allocating health care resources to improve people's health, those who have adapted the most should receive lower priority in healthcare as a result of the laudable effort associated with coming to terms with their condition – or whether we should accept more experienced suffering in the world in order to reward those who expended effort in adapting to their condition. This study cannot resolve this “vexing moral problem” (Murray, 1996) and interested readers should see Menzel et al. (2002) for an overview of the ethical issues.

What the study does do, however, is shed light on the importance of a related normative debate – namely, the degree to which preferences should be “informed desires” (Harsanyi, 1985). Those who favour a preference satisfaction account of welfare generally argue that those preferences should include “information regarding the experiential quality of some feature of outcomes” (Adler, 2012, p. 214). Insofar as information about the satisfaction level associated with a health state is seen as relevant additional information, we have shown that “better informed” preferences are different from “less informed” ones.

In the very least, therefore, future studies should consider the impact on health state preferences of different types and levels of information about the future experiences of those preferences. In this way, empirical data can illuminate the debate about what sort of preferences (if indeed any) should be used to value health and other benefits.

## Figures and Tables

**Table 1:** Versions and states used in scenario A.

Version 1	Version 2	Version 3
Unable to walk and high LS	Unable to walk and learnt to live	Unable to walk and high HS
Extreme pain and learnt to live	Unable to walk and low HS	
Extreme pain and low HS	Extreme pain and high LS	
Extreme depression and high HS	Extreme pain and high HS	
	Extreme depression and learnt to live	

No. of respondents = 213	=211	=221
No. of observations = 852	=1055	=221

*Note*: LS and HS denote life satisfaction and health satisfaction, respectively. No. of observations is ‘No. of respondents × number of states in version’.

**Table 2:** Descriptive statistics.

	Mean	Own health in EQ-5D-5L	Mean
Age	42.2		
Male	43.1	Mobility level 1	72.6
		Mobility level 2	14
*Marital status*		Mobility level 3	7
Married	57.4	Mobility level 4	5.4
Separated	2.2	Mobility level 5	0.9
Divorced	9.6	Self-care level 1	87.3
Widowed	1.7	Self-care level 2	6.6
		Self-care level 3	4.7
*Employment status*		Self-care level 4	0.9
Employed	53.3	Self-care level 5	0.5
Retired	8.4	Usual activities level 1	71
Taking care of home	9.3	Usual activities level 2	13.9
Student	8.1	Usual activities level 3	10
Seeking work	4.7	Usual activities level 4	4.4
Unemployed	5.9	Usual activities level 5	0.6
Long-term sick	8.4	Pain/discomfort level 1	45.2
		Pain/discomfort level 2	31.7
*Education level*		Pain/discomfort level 3	15.3
Degree level	38.8	Pain/discomfort level 4	5.8
Below degree level	36	Pain/discomfort level 5	2
		Anxiety/depression level 1	55.7
*SWB*		Anxiety/depression level 2	23.6
Satisfaction with health (*SWB*_H_)	6.4	Anxiety/depression level 3	13.3
Low (0–5)	33.3	Anxiety/depression level 4	5
Medium (6–7)	26.1	Anxiety/depression level 5	2.5
High (8–9)	30.7		
Very high (10)	9.9	Full health	31.6
Satisfaction with life (*SWB*_L_)	6.3		
Low (0–5)	34.4		
Medium (6–7)	29.6		
High (8–9)	26.7		
Very high (10)	9.3		

*Note*: For the case of binary variables, means represent proportions (%).

**Table 3:** Proportion of respondents choosing full health scenario (scenario B).

Unable to walk and high LS	53.1%
Unable to walk and high HS	56.6%
Unable to walk and learnt to live	58.3%
Extreme depression and learnt to live	70.6%
Extreme depression and high HS	70.9%
Extreme pain and high LS	71.1%
Extreme pain and learnt to live	71.8%
Extreme pain and high HS	73%
Unable to walk and low HS	86.3%
Extreme pain and low HS	90.1%

**Table 4:** Regressions grouped by health state.

	(1) ‘Unable to Walk’	(2) ‘Extreme pain’	(3) ‘Extreme depression’
*Health scenarios*			
Unable to walk and low HS	0.23** (0.043)		
Unable to walk and high HS	−0.121* (0.056)		
Unable to walk and high LS	−0.144* (0.057)		
Unable to walk and learnt to live	−0.096 (0.056)		
Unable to walk	Reference group		

Extreme pain and low HS		−0.071 (0.045)	
Extreme pain and high HS		−0.292** (0.051)	
Extreme pain and high LS		−0.316** (0.052)	
Extreme pain and learnt to live		−0.322** (0.052)	
Extreme pain		Reference group	

Extreme depression and high HS			−0.305** (0.051)
Extreme depression and learnt to live			−0.301** (0.044)
Extreme depression			Reference group
*Demographics*			
Age		0.018* (0.007)	
Age^2^		−0.0002* (0.0001)	
Employment: retired		0.076* (0.037)	
Employment: long-term sick		0.076* (0.037)	
Employment: taking care of home			−0.15* (0.068)
Education: below degree level		0.06* (0.03)	0.083* (0.041)
*Own health in EQ-5D-5L*			
Mobility level 5		0.126** (0.034)	
Usual activities level 2			0.104* (0.046)
Pain/discomfort level 2			−0.09* (0.045)
Pain/discomfort level 3		−0.134* (0.062)	
Anxiety/depression level 2			−0.106* (0.051)
Anxiety/depression level 3	−0.14* (0.062)	−0.193** (0.059)	−0.162* (0.074)
Anxiety/depression level 4	−0.228** (0.087)		−0.26* (0.111)

Demographics	Yes	Yes	Yes
EQ-5D-5L	Yes	Yes	Yes
*SWB*_L_	Yes	Yes	Yes

*N*	972	1211	613
Pseudo-*R*^2^	0.094	0.173	0.164

*Note*: Regressions are probits. Dependent variable is the binary variable of choosing full health for three years. Coefficients are marginal effects. Robust standard errors clustered at the individual level within parentheses. Demographic base categories are: being single (marital status), employed (employment status), having no degree (education), low *SWB*_L_, and all level 1 severities for EQ-5D-5L dimensions.

* *p* < 0.05.

** *p* < 0.01.

**Table A1:** EQ-5D-5L and EQ-5D-3L.

		EQ-5D-5L	EQ-5D-3L
Mobility	1.	I have no problems in walking about	I have no problems in walking about
	2.	I have slight problems in walking about	I have some problems in walking about
	3.	I have moderate problems in walking about	I am confined to bed
	4.	I have severe problems in walking about	
	5.	I am unable to walk about	

Self-care	1.	I have no problems washing or dressing myself	I have no problems with self-care
	2.	I have slight problems washing or dressing myself	I have some problems washing or dressing myself
	3.	I have moderate problems washing or dressing myself	I am unable to wash or dress myself
	4.	I have severe problems washing or dressing myself	
	5.	I am unable to wash or dress myself	

Usual activities	1.	I have no problems doing my usual activities	I have no problems with performing my usual activities
	2.	I have slight problems doing my usual activities	I have some problems with performing my usual activities
	3.	I have moderate problems doing my usual activities	I am unable to perform my usual activities
	4.	I have severe problems doing my usual activities	
	5.	I am unable to do my usual activities	

Pain/discomfort	1.	I have no pain or discomfort	I have no pain or discomfort
	2.	I have slight pain or discomfort	I have moderate pain or discomfort
	3.	I have moderate pain or discomfort	I have extreme pain or discomfort
	4.	I have severe pain or discomfort	
	5.	I have extreme pain or discomfort	

Anxiety/depression	1.	I am not anxious or depressed	I am not anxious or depressed
	2.	I am slightly anxious or depressed	I am moderately anxious or depressed
	3.	I am moderately anxious or depressed	I am extremely anxious or depressed
	4.	I am severely anxious or depressed	
	5.	I am extremely anxious or depressed	
